# How the Brain Homes in on Valuable Objects

**DOI:** 10.1371/journal.pbio.1001228

**Published:** 2011-12-27

**Authors:** Janelle Weaver

**Affiliations:** Freelance Science Writer, Glenwood Springs, Colorado, United States of America

**Figure pbio-1001228-g001:**
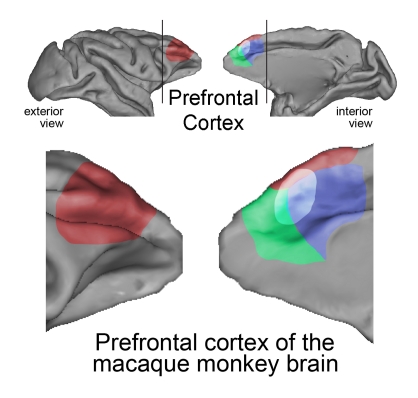
Three major subdivisions (colored) of the primate prefrontal cortex contain neurons guiding the focus of attention. Neurons at the intersection of these subdivisions (shaded) carry information about both the value and location of objects.

[Fig pbio-1001228-g001]Our visual surroundings are often crowded with information, and we must focus on the most crucial details to avoid being overwhelmed. The brain automatically detects and biases attention toward important and valuable features, such as the green flash of a traffic light and the shape and color of a dollar bill lying on the sidewalk. Although scientists have identified brain regions involved in either shifting attention or assessing the value of items, relatively little is known about the interplay between these regions.

In a paper published this week in *PLoS Biology*, a team led by Thilo Womelsdorf of York University in Toronto, Canada, addressed this issue by recording from neurons across a large portion of the monkey brain. They found that two regions—the ventromedial prefrontal cortex (vmPFC) and the anterior cingulate cortex (ACC)—are involved in guiding attention to the most valuable objects in a scene. The findings provide a more complete understanding of the roles of different brain areas and their interactions during this crucial task.

In the study, the researchers trained two monkeys to perform a visual attention task. The animals fixated on a gray dot in the center of a screen and saw a circle filled with red and black stripes on one side and another circle with green and black stripes on the other side. Next, the color of the dot changed to red or green to cue the monkeys to pay attention to the corresponding pattern, which then rotated either clockwise or counterclockwise. The animals indicated the direction of motion by moving their eyes up or down, respectively, and they received juice for correct decisions. But the amount of the reward depended on the color of the pattern, and accuracy was higher for stimuli linked to a larger reward than for those associated with less juice.

While the monkeys were engaged in the task, the researchers recorded from more than 1,000 neurons in the frontal and cingulate cortex. The activity of some neurons carried precise information about the location of the attended pattern, and these cells were clustered in the ACC, vmPFC, and lateral prefrontal cortex. A subset of these neurons showed transient changes in their firing rates immediately after the dot changed color, suggesting that they help to shift attention to task-relevant stimuli. By contrast, a separate set of ACC neurons showing delayed and prolonged responses could play a role in maintaining attention to relevant locations while filtering out distractions.

The research team also examined how neuronal activity was affected by reward magnitude. Neurons that were sensitive to the value of the attended pattern formed a distinct set of clusters in the vmPFC and ACC, but some cells in these two regions responded to both the location and value of the stimulus. Because the ACC is connected with brain areas involved in directing eye gaze and planning other movements, and the vmPFC shares connections with areas that are critical for memory and emotions, these two regions are ideally positioned to integrate information about location and value to focus attention on the most important objects in the environment.

**Kaping D, Vinck M, Hutchison RM, Everling S, Womelsdorf T (2011) Specific Contributions of Ventromedial, Anterior Cingulate, and Lateral Prefrontal Cortex for Attentional Selection and Stimulus Valuation. doi:10.1371/journal.pbio.1001224**

